# Study on the Effects of Salt Tolerance Type, Soil Salinity and Soil Characteristics on the Element Composition of Chenopodiaceae Halophytes

**DOI:** 10.3390/plants11101288

**Published:** 2022-05-11

**Authors:** Xiaoqian Song, Yuhang Su, Jingwen Zheng, Zhonghua Zhang, Zhengwei Liang, Zhonghua Tang

**Affiliations:** 1 The Key Laboratory of Forest Plant Ecology, Northeast Forestry University, Harbin 150040, China; sxq_824096061@163.com (X.S.); syh1832264776@163.com (Y.S.); zjw06220518@163.com (J.Z.); 2 College of Chemistry, Chemical Engineer and Resource Utilization, Northeast Forestry University, Harbin 150040, China; 3 Northeast Institute of Geography and Agroecology, Chinese Academy of Sciences, Changchun 130102, China; liangzw@neigae.ac.cn

**Keywords:** halophytic, environment, genetic, element, composition

## Abstract

With the continuous increase in saline–alkali land, sustainable development of the global environment and ecology have been seriously affected. This study compared the absorption and accumulation patterns of 11 elements in different parts (roots, stems and leaves) of different leaf Na regulation strategies of the pioneer plant Chenopodiaceae in saline–alkali land and evaluated the effects of soil nutrient status and soil salinity on the distribution of plant elements. The results showed that the changes in the content of Ca, Mg and Na in plants are affected by the salt-tolerant type and on different parts. Soil salinity had no significant effect on element concentrations in different parts of plants. The Pearson correlation coefficient showed that the correlation between plants and soil elements was different, and different parts of plants had the characteristics of selective absorption of soil elements. The salt tolerance type and soil mineral element concentrations explained most of the variation observed in element concentrations in Chenopodiaceae plants; the soil salinity property played only a minor role. It was concluded that the genetic factors are the prerequisite in the composition pattern of leaf elements in Chenopodiaceae, and soil factors are the key to determining element accumulation. These conclusions provide an effective reference for evaluating plant breeding and its response to environmental change in saline–alkali arid areas in Hulunbuir grassland and other parts of the world.

## 1. Introduction

Soil salinization is a worldwide problem in terms of resources and ecology [1]. Currently, approximately 20% of the irrigated soil in the world is affected by salinity, which continues to deteriorate [2]. It is estimated that by 2050, more than 50% of cultivated land will be salinized [3,4], which will be a severe threat to land utilization rate and crop yield [5]. Soil salinization decreases soil nutrient availability [6] and affects the nutrient supply to plants and the absorption and utilization of nutrients by plants [7]. Soil nutrients are among the most influential drivers of species distribution, morphological characteristics and biomass production [8]. In general, plants require maintenance of mineral element concentrations within specific ranges for optimum growth and functioning [9]. Changes in the availability of soil nutrients will affect the composition of plant elements, thereby affecting the optimal state of plant growth and function [10]. Many previous studies have focused on the survival of halophytes [11], whereas halophytes growing in saline–alkali environments have received limited attention in terms of nutrient resorption and element composition strategies. A better understanding of nutrient uptake and allocation in halophytes, as well as their relationships with environmental conditions, is crucial for predicting how saline–alkali ecosystems respond to seasonal salt accumulation change.

Halophyte species are rare, and only 0.25% of angiosperm species can complete their life history in a saline environment [12]. Halophytes exhibit various adaptive strategies to salinity [13]. According to the physiological adaptation to salinity, it is divided into three main types: salt-dilution halophyte, recretohalophyte and pseudohalophyte [14]. The family Chenopodiaceae is one of the most successful plant groups adapted to high salinity and drought conditions [15]. Some of these species not only tolerate high levels of salinity, but display optimal growth under saline conditions [16]. These indicate that the Chenopodiaceae plant has abundant nutrient absorption and allocation strategies in order to sustain high levels of photosynthesis and rapid growth in saline environments. This family mainly includes both salt-dilution halophyte and recretohalophyte [17]. The aboveground parts of salt-dilution halophytes become fleshy, which can absorb more water, dilute the salt absorbed into the plant, and reduce the salt concentration in the cells to a concentration that will not cause harm [14]. At the same time, salt is accumulated in the fleshy tissues of the leaves or stems and the vacuoles of the green tissues, which reduces the toxicity of the salt in the leaves, assimilated stems, or both [18]. The recretohalophyte have special salt-secreting structure-salt glands or salt vesicles, which can excrete excessive salt in the plant body into the body. A salt vesicle is a special kind of salt gland. Its particularity is that the salt it secretes is not excreted from the body but accumulates in a vesicular cell. Salt vesicles exist in some genera of Chenopodiaceae, such as Atriplex, all species of Atriplex, and most species of Chenopodium [19]. However, there is still no good understanding about nutrient utilization strategies of different adaptive strategies.

Halophytes not only face limiting nutrient absorption, but also face ion toxic effects in the salt environment. The salt tolerance of halophytes depends on the ability of plants to eliminate Na and maintain the nutrient balance in the body [20]. To a certain extent, halophytes increase the accumulation of Na and decrease the accumulation of K, Ca and Mg under salt stress [21]. However, there are also experiments that show that as the salinity increases, K, Ca, and Mg either increase or remain unchanged [22]. Some experiments have shown that different halophytes also adapt to and resist the salt environment through changes in the distribution of elements in different organs. In recent years, some scholars have conducted laboratory experiments on the nutrient distribution of the halophytes *Suaeda glauca* and *Salicornia europaea* in the saline environment and how they adapt to salt changes. They found that as the salinity increases, the Na concentration in all organs of *S. glauca* and *S. europaea* increases significantly. Conversely, K, Ca and Mg concentrations in leaves of *S. glauca* and stems of *S. europaea* significantly decreased with increasing salinity. Moreover, changes in soil concentration can also have various nutrient absorption strategies. *S. glauca* grew best in 0.1 mol·L^−1^ salt in soil, whereas 0.2 mol·l^−1^ salt in the soil was most appropriate for *S. europaea* [10]. This phenomenon was also noted in a study of the less salt-tolerant species *Sonneratia lanceolata*, which showed a clear preference for low salinities, and the more salt tolerant *S. alba* grew fastest at considerably higher concentrations [23]. Other studies found that the growth of *Acacia auriculiformis* and olive significantly decreased with increases in soil salinity [24,25]. These contradictory results could arise from changes in the element utilization strategies of different salt-tolerant types of plants to the environment.

Understanding the relationship between nutrient element components and the salt environment has important guiding significance for studying how plants adapt and survive under salt environment conditions. However, as far as we know, there are relatively few studies on the relationship between the element components of different salt-tolerant types of halophytes and the natural salt environment. In this study, we analyzed 11 elements in different plant organs and the rhizosphere of soil, which included eight Chenopodiaceae species in the Hulunbuir grassland, China. The following issues were scrutinized and explored through this study: it was hypothesized that the element concentrations of halophytes are primarily affected by genes (according to different salt tolerance types) and soil mineral elements in salt environments, whereas they are less affected by salt and remain relatively stable under the change of salt concentration. Verification: (1) The connection between salt concentration and plant element concentration is weak. (2) Salt tolerance types are related to the concentration of plant elements. (3) Soil mineral elements have a significant influence on the concentration of plant elements.

## 2. Results

### 2.1. Patterns of Element Concentrations in the Leaf, Stem and Root

Element concentrations showed considerable variation among plant tissue parts (leaves, stems and roots) and salt tolerance types (Table 1). In salt-dilution halophyte, Na had a higher level of accumulation in the leaves. Compared with the root and stem, Na accumulation was approximately 200% in the salt-dilution halophyte leaves. In recretohalophytes, the elements of K, Ca and Mg, had a higher level of accumulation in recretohalophytes leaves. Compared with the root and stem, K, Ca and Mg increased approximately 20%, 60% and 60% in the leaves, respectively. In addition, C was found to accumulate more in the roots than in the leaves and stems in salt-diluted halophyte and recretohalophytes.

The results of ANOVA showed various effects of plant parts, salt tolerance type and their relationship with element concentrations (Table 2). Salt tolerance type had significant effects on K, Ca, Mg, Na, Mn and Na/K concentrations in plants. The plant part also had a significant effect on the concentrations of P, N, C, Ca, Mg, Na and Mn. It was found that under the interaction of different salt-tolerant types and different parts of plants, all elements are not significantly different, indicating that the changes in plant elements are not affected by the common influence of salt-tolerant types and parts. The results showed that the photosynthesis related elements (i.e., N, P, Mg and K) revealed higher concentrations in recretohalophytes than in salt-dilution halophytes. However, Na showed the highest concentrations in the leaves, stems and roots of salt-dilution halophytes, as did Na/K (Table 1).

### 2.2. Relationships between Element Concentrations of Halophytes and Soil Salinity

**Figure 1:**
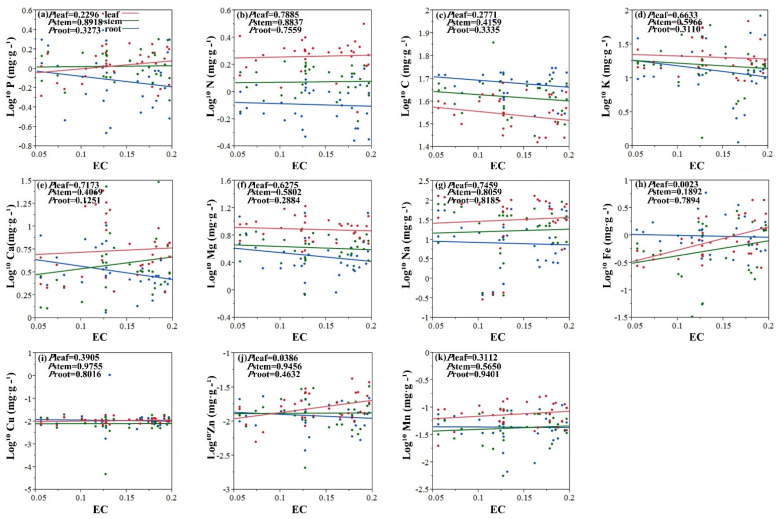
Linear relationships of EC (dS m^−1^) with concentration of 11 elements among leaves, stems and roots. Colored lines represent significant relationships (*p* < 0.05) for halophytes (red, leaves; blue, stems; green, roots). (**a**) Linear relationship between log 10 P (mg g^−1^) and EC. (**b**) Linear relationship between log 10 N (mg g^−1^) and EC. (**c**) Linear relationship between log 10 C (mg g^−1^) and EC. (**d**) Linear relationship between log 10 K (mg g^−1^) and EC. (**e**) Linear relationship between log 10 Ca (mg g^−1^) and EC. (**f**) Linear relationship between log 10 Mg (mg g^−1^) and EC. (**g**) Linear relationship between log 10 Na (mg g^−1^) and EC. (**h**) Linear relationship between log 10 Fe (mg g^−1^) and EC. (**i**) Linear relationship between log 10 Cu (mg g^−1^) and EC. (**j**) Linear relationship between log 10 Zn (mg g^−1^) and EC. (**k**) Linear relationship between log 10 Mn (mg g^−1^) and EC.

**Figure 2:**
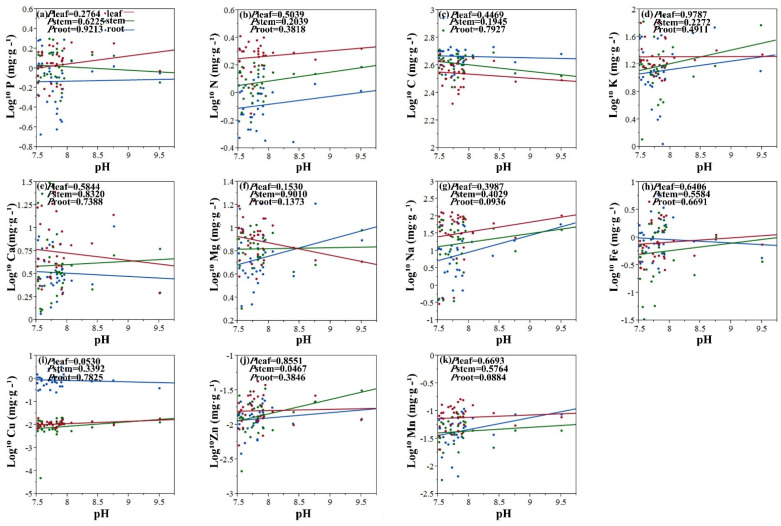
Linear relationships of pH with concentration of 11 elements among leaves, stems and roots. Colored lines represent significant relationships (*p* < 0.05) for halophytes (red, leaves; blue, stems; green, roots). (**a**) Linear relationship between log 10 P (mg g^−1^) and pH. (**b**) Linear relationship between log 10 N (mg g^−1^) and pH. (**c**) Linear relationship between log 10 C (mg g^−1^) and pH. (**d**) Linear relationship between log 10 K (mg g^−1^) and pH. (**e**) Linear relationship between log 10 Ca (mg g^−1^) and pH. (**f**) Linear relationship between log 10 Mg (mg g^−1^) and pH. (**g**) Linear relationship between log 10 Na (mg g^−1^) and pH. (**h**) Linear relationship between log 10 Fe (mg g^−1^) and pH. (**i**) Linear relationship between log 10 Cu (mg g^−1^) and pH. (**j**) Linear relationship between log 10 Zn (mg g^−1^) and pH. (**k**) Linear relationship between log 10 Mn (mg g^−1^) and pH.

The results show that there was significant correlation between Fe, Zn concentrations in leaf and the EC (Figure 1h,j). In conclusion, pH value has a remarkable influence on the concentration of Zn in plant stems (Figure 2j).

### 2.3. Correlations between Element Concentrations of Halophytes and Soil Mineral Elements

**Figure 3:**
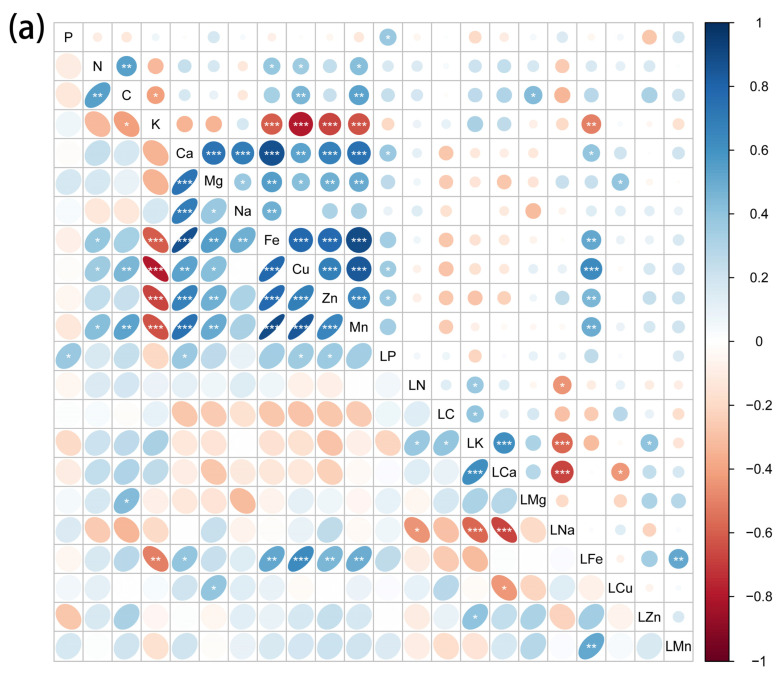

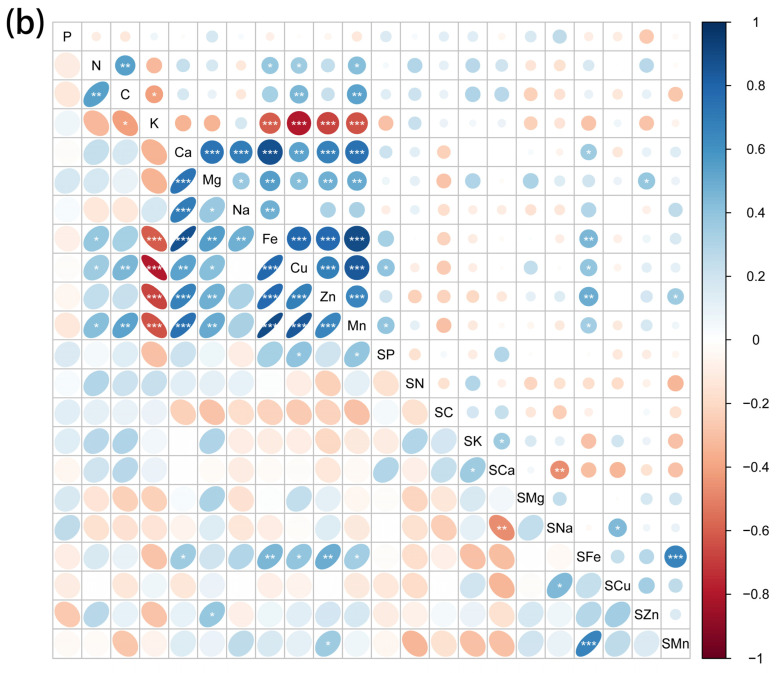

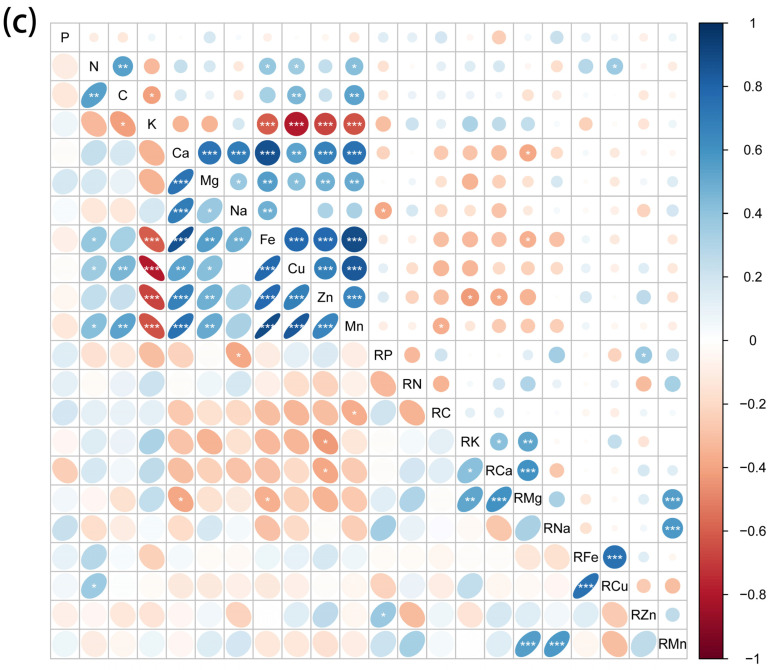
Pearson correlations between leaf (**a**), stem (**b**) and root (**c**) minerals and soil nutrients. Leaf, stem and root minerals (mg g^−^^1^) were log 10 transformed before analysis. Soil elements are content based (mg g^−^^1^). “*”, “**” and “***” indicate that the difference is significant (*p* < 0.05), highly significant (*p* < 0.01) and very significant (*p* < 0.001).

Element concentrations in leaves, stems and roots showed different correlations with soil element concentrations (Figure 3). The Pearson correlation coefficients showed that leaf P was positively correlated with soil P, Ca, Cu and Zn; leaf Mg was positively correlated with soil C; leaf Fe was positively correlated with soil K, Ca, Fe, Cu, Zn, and Mn; and leaf Cu was positively correlated with soil Na. Stem P was positively correlated with soil Cu and Mn; stem Fe was positively correlated with soil Ca, Fe, Cu, Zn, and Mn; and stem Zn was positively correlated with soil Mg. Toot P was positively correlated with soil Na;, and root C was positively correlated with soil Mn. Soil Zn was positively correlated with root K and Ca, root Mg was positively correlated with soil Ca and Fe, and root Cu was positively correlated with soil N. In addition, it was also found that there is a strong autocorrelation of mineral elements in the soil.

### 2.4. Relationship between the Elemental Composition of Plants, Soil Mineral Elements, and Soil Salinity

**Figure 4:**
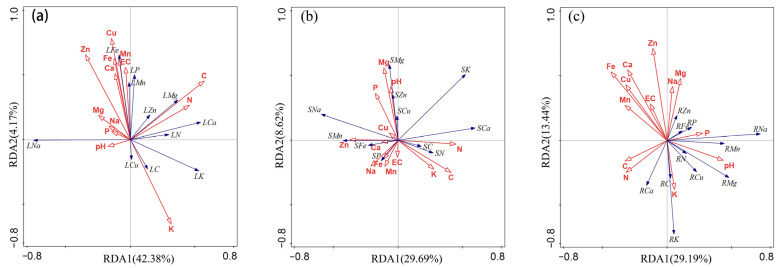
RDA analysis of the soil mineral elements and soil salt vs. plant leaf (**a**), stem (**b**) and root (**c**) mineral elements.

Redundant analysis of mineral elements in different parts of plants, soil mineral elements, and soil salinity were carried out (Figure 4). According to the RDA analyses, plant leaves showed high Na concentrations (Figure 4a). Soil C was the largest influencing factor of mineral elements in plant leaves, with an explanation rate of 14.8% (F = 5.2, *p* = 0.01), and it showed a positive correlation with Mg in leaves. Plant stems showed high K and Na concentrations (Figure 4b). The Mg and pH in the soil were correlated with the Mg in the stem. Plants root showed high K and Na concentrations (Figure 4c). Soil Fe and Zn were the largest influencing factor of mineral elements in plant roots, with an explanation rate of 9.6% (F = 5.2, *p* = 0.05), and there was a strong negative correlation between K in soil and K in roots, and the Mg in the soil had a strong negative correlation with the Mg in the root.

### 2.5. Element Composition in the Leaf, Stem and Root and Environmental Control

The general linear model (GLM) was employed to model the effect of salt tolerance type, soil salinity properties and soil mineral elements on halophyte element concentrations. According to the results, it was discovered that the obtained models altogether explain a substantial part of the variance in element concentrations of plant leaves (Table 3), stems (Table S1) and roots (Table S2). For leaf elements, the model accounts for 26–69% of the total variability, where salt tolerance type and soil mineral elements explain 0.01–17% and 19–55% of the total variances, respectively; but salt only explains 0.5–10% of the variances. In addition, there are various conclusions that can be drawn from these three factors. For example, soil mineral elements account for most of the variances among all elements (Table 3). For stem elements, the model accounted for 35–68% of the total variability, wherein salt tolerance type and soil mineral element factors explain 0.01–26% and 22–58% of the total variances, respectively, but salt only explains 0.3–16% of the variances (Table S1). For root elements, the models reveal more significance than that of the leaf and stem, accounting for 35–70% of the total variation. More of the variances are explained by soil mineral element factors (0.03–47.6%) than by salt tolerance type (20–52%). In addition, soil mineral element factors explain more of the total variances of other elements but calcium (Table S2).

## 3. Discussion

Some studies have found that the concentration of plant elements in arid environments is affected by the classification of plant and soil mineral elements [26,27,28]. However, the effects of salt tolerance type and the salt environment on the concentration of elements in different plant organs have not been resolved. The analysis carried out in this study provides new insights into the relationships between element concentrations in different plant parts and salt tolerance type, salt stress and soil mineral elements.

### 3.1. The Effect of Salt Tolerance Type on Element Concentrations in Different Plant Parts

Plant parts and salt tolerance type had varied effects on element concentrations. The variance components attributable to salt tolerance type and part differed considerably between elements. This relationship is mainly driven by elements directly related to salinity, such as Na, Mg, K, and Ca [29]. This paper found that the content of Ca, Mg, and Na in plants is significantly different from the salt-tolerant type and is significantly different from different parts, indicating that the changes in the content of Ca, Mg and Na in plants are affected by the salt-tolerant type and different parts, but we found that the change in plant element content was not affected by the combined action of these two factors.

This paper found that salt-dilution halophytes showed higher concentrations of Na than in recretohalophytes; the content of K, Ca and Mg was lower than that of recretohalophytes. Na is necessary for osmotic adjustment and for the maintenance of optimum growth in halophytes [30]. In saline soils, a high concentration of Na in plants saturate the binding sites of ion pumps in the saturating salt-tolerance pathway, resulting in reduced potassium absorption [31]. Several researchers have confirmed that halophytes that grow in saline–alkali environments, such as *Suaeda maritima* [32] and *Sarcobatus vermiculatus* [33], can take advantage of Na to develop some key functions instead of K [34]. Therefore, compared with the recretohalophytes, the salt-diluted halophytes accumulate a higher concentration of Na, thereby reducing the K concentration. In addition, salt ions in salt-dilution halophytes accumulate in the vacuoles of succulent leaf tissues and other green tissues, as well as in the succulent column. Contrastingly, recretohalophytes have the ability to secrete salt through specialized leaf structures (salt glands), which is arguably one of the most remarkable features of recretohalophytes [35]. This may explain why we observed slightly lower Na concentration in the leaves of recretohalophytes than in salt-dilution halophytes. Ca regulates Na^+^/H^+^ reverse transportation in vacuoles as well as inhibits the entry of Na into roots [29]. It was observed in this study that the high concentration of Ca in recretohalophytes is likely to be associated with its increase, leading to the downregulation of Na absorption. Na accumulation along with Mg in leaves of halophytes plays an important role in osmotic regulation and water uptake in saline soils [36]. In our work, it was found that the Mg content of salt-diluted halophytes (7.41 ± 0.3 mg·g^−1^) and recretohalophytes (9.75 ± 0.4 mg·g^−1^) was higher than the Mg content required by higher plants (2.0 mg g^−1^), the reason is that Mg plays an important role in photosynthesis, membrane transport and enzyme activation [37] and preferentially combines with N and P groups. Mg in leaves can provide high photosynthesis for fast-growing halophytes [38]. The accumulation of high Mg concentration in the leaves is conducive to regulating the growth of plants in a saline–alkali environment. Therefore, this result is related to the different roles of mineral elements in different salt tolerance types.

This paper found that the changes in N, C and Na content in halophytes were affected by different parts. Studies have found that in salt-diluted halophytes and recretohalophytes, with the exception of C, other elements accumulate more in the leaves than in the stems and roots. The main reason for this result is that leaves are the most sensitive organs in response to environmental changes [39]. High salt stress in saline soil will cause stomata to close, restrict gas diffusion and lead to hypoxia in the roots, and the photosynthesis is inhibited, but dry matter accumulation is reduced [40], resulting in lower C content in the leaves of halophytes. Leaves are also the main organ that accumulates N. During the sampling period, plants have strong photosynthesis and strong transpiration, and they absorb more N to maintain normal physiological functions. The N absorbed by the roots is transported upward to the leaves with the water, such that the N element accumulates in the leaves; thus, the N element of the leaves is preferentially distributed. Some halophytes species, such as Phragmites australis, can recirculate Na from the shoots back to the roots, keeping low Na concentrations in shoot vacuoles and high Na in root vacuoles [27,41]. Therefore, changes in the chemical composition of halophytes are not only related to the type of salt tolerance, but are also related to the distribution of nutrients in different parts and to the selective absorption of different elements.

### 3.2. The Effects of Soil Salinity on Element Concentrations in Different Plant Parts

The EC is often used to indicate the level of soil salinity [42]. The pH value is an important chemical property of the soil. EC and pH value were used as salt stress factors. This paper found that there was a significant effect of pH value and EC on the concentration of Zn in leaf and stem. There was significant correlation between Fe concentrations in leaf and the EC. It could be argued that the pH value and EC reveals the same plant element correlation trends. This reflects the plant’s physiological and biochemical external conditions of the environment [43]. The soil type in this studied area is calcareous chernozem, in which the availability of N and P is relatively low. There are reports that the ionome of Arabidopsis thaliana plants grown under phosphorus-deficient environment revealed significantly increased concentrations of Fe and Zn [44], which further prove the interaction among mineral element homeostasis in plants. As the functions of different plant organs are different, internal stability of different organs tends to vary in the same species [45]. For example, in the degraded grasslands of Northeast China, the internal stability of N and P in the roots of *Leymus chinensis* (Trin.) Tzvel was higher than that in the leaves [46]. Studies on tree seedlings and shrub plants have also found that the internal stoichiometric stability of plant leaves is higher than that of roots [47]. It can be seen from the results that the elemental internal stability of halophytes leaves is also higher than that of roots. Therefore, plants maintain their own growth and development needs by coordinating the nutrient distribution among various organs in a saline–alkali and arid environment.

### 3.3. Element Composition in the Leaf, Stem and Root and Environmental Control

The discoveries in this study support our hypothesis that soil mineral elements are more important than salt tolerance type and salt property in explaining the variation of element concentrations across halophytes. According to the results of the partial GLMs, it was found that the percentage of elemental variations explained by the three factors combined (salt tolerance type, soil salinity and soil mineral elements) varied among different organs: 26% to 69% of the variation was explained for leaves (Table 3), 35% to 68% for stem (Table S2), and 35% to 70% for roots (Table S3). For leaf, stem and root elements, soil mineral elements had greater explanatory power. The productivity, functioning and biogeochemical cycles of terrestrial ecosystems are strongly affected by leaf element concentrations [28]. Soil mineral elements and salt tolerance type explained most of the variation in element concentrations in Chenopodiaceae (Amaranthaceae). Out of the three factors, soil properties explained most of the variances in 11 leaf elements (Table 3). Since the availability of these elements largely depends on soil water conditions, and the soil of the study area is arid and calcareous, where K, Mn, Zn, Cu, and Fe mainly exist in non-exchangeable forms, these elements are largely in limited supply to the halophytes. Similar to leaf element concentrations, salt tolerance type and soil mineral elements explained more of the variance in stem and root elements than soil salinity (Tables S2 and S3). Several studies have shown that the accumulation of certain elements in the leaves of halophytes is genetically controlled as primarily independent on the condition of their rhizospheres [19,48]. However, some studies have put forward a different point of view, pointing out that most of the mineral elements found in plant tissues come from the soil. The variations in these element concentrations across desert shrubs are mainly explained by soil characteristics [30]. The reason for this situation may be related to the research environment. The low content of soil nutrients in deserts and saline–alkali environments can promote plants to accumulate more elements to maintain normal growth. Thus, necessitating plants to adapt to highly variable soil compositions to survive and thrive. To adapt to the instability of soil element availability, halophytes must alter the uptake and storage of both nutrients as well as elements. Therefore, it is suggested that genetic factors are the prerequisite in the composition pattern of leaf elements in Chenopodiaceae, while soil factors are the key to determining element accumulation.

## 4. Materials and Methods

### 4.1. Study Area and Species

**Figure 5:**
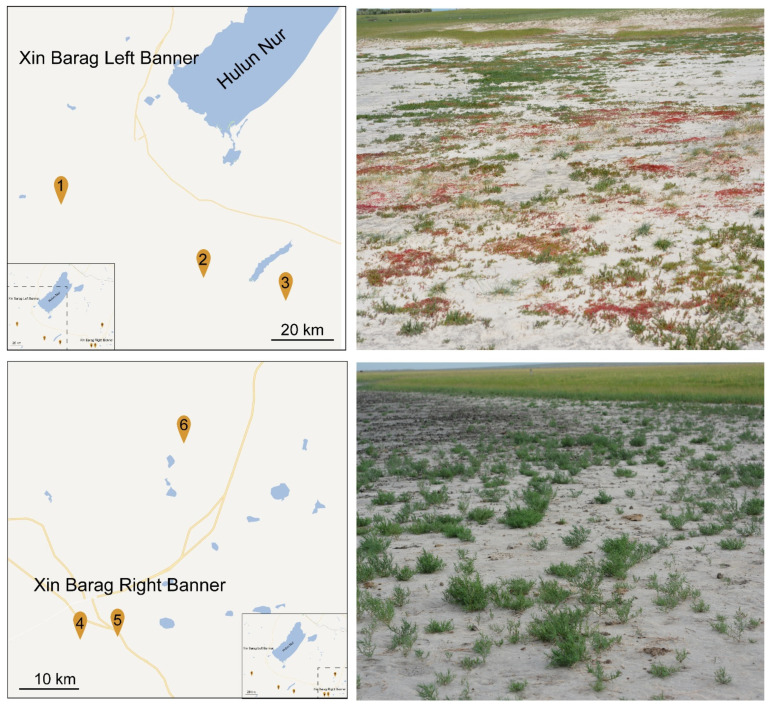
Location of the studied sites for the studied locations, and pictures of the vegetation on site.

From 2016 to 2017, the germplasm resources of saline-tolerant plants in the saline–alkali land of Hulunbuir grassland in Inner Mongolia were surveyed, and the Chenopodiaceae with higher species richness was finally determined. We collected 19 species of Chenopodiaceae in total. There are only eight known salt-tolerant types, five of which are salt-diluted halophytes and three that are halophytes that secrete halophytes. The research area includes Xin Barag Left Banner and Xin Barag Right Banner. Three plots were set up in Xin Barag Left Banner, and three plots were set up in Xin Barag Right Banner (Figure 5; Table 4).

Xin Barag Left Banner is the largest soil saline–alkali land in Hulunbuir Grassland, and its salinization area accounts for 71% of the total saline–alkali area. Xin Barag Right Banner is the second largest soil saline–alkali land in Hulunbuir Grassland, and its salinization area accounts for 14% of the total saline–alkali area. The climates of these two regions are similar, and both belong to the mid-temperate continental monsoon climate, with long and severe winters and a snow cover period of about 140 days. The annual average temperature is 0.2 °C, the annual precipitation is about 280 mm, and the annual evaporation is 950 to 1900 mm. Its complex natural environment constitutes a natural grassland halotype. Among them, the halophytes mainly include *Suaeda salsa*, *Suaeda salsa*, *Atriplex siberia*, *Kochia scoparia*, etc., which are distributed on the shores of saline lakes, deserted beaches and sandy grasslands. Due to the low precipitation and large evaporation, the soil salt-alkali components are deposited, and the soil parent material has a high salt content.

In this study, plant samples (taken from the early stage of plant growth) and the soil around the roots were collected from 27 June to 3 July 2018. Ten well-growing plants of the same size from each plant in each plot were collected. The same plants in different places were regarded as duplicates, and there were 4 duplicates in total. We collected a total of 32 samples from 8 plant species for chemical analysis. In addition, we collected 0–20 cm of uniform soil around the plant roots (Table 5). Among them, the plant materials were identified by Professor Mu Liqiang from Northeast Forestry University. The germplasm and DNA materials of the voucher samples are stored in The Germplasm Bank of Wild Species (http://www.genobank.org (accessed on 1 August 2016)), and the numbering information is shown in Additional file 1.

### 4.2. Soil and Plant Sampling and Chemical Analysis

Aboveground and belowground parts of the plants were kept intact during sample collection. The collected plants were stored in a fresh-keeping box and taken back to the laboratory. The leaf, stem and root samples were rinsed twice with deionized water to remove dust and soil. They were then oven-dried at 120 °C for 20 min, cooled to 60 °C and desiccated for 48 h. Soil samples were air-dried, crushed, mixed and sieved through a 2-mm sieve before use.

For each plant sample, 0.2 g portion of prepared product was weighed and added into a PTFE beaker along with 5 mL nitric acid as well as 1 mL perchloric acid. The sample was digested using microwave digestion apparatus (MARSXpress, CEM, Matthews, NC, USA). The digestion temperature was 160 °C for 120 min and 200 °C until nearly dry. The sample was processed until turning white and transparent, and the liquid in the tube was clear, with approximately 0.5 mL of liquid remaining. After the digestion was complete, and the digestion tube had been cooled, the volume of the plant sample was fixed with 2% nitric acid to 25 mL.

The soil digestion procedure was the same as the plant digestion procedure, except for the volume of acid added. A 0.2 g soil sample was added in 5 mL nitric acid, 5 mL hydrofluoric acid and 2 mL perchloric acid for digestion; the sample was fixed with 2% nitric acid to 50 mL.

Blank solutions (acid mixture without samples) were measured in duplicate during each group of sample digestions. The concentrations in the plant and soil samples of the elements K, Ca, Na, Mg, Mn, Cu, Zn and Fe were determined using inductively coupled plasma optical emission spectrometry (ICP-OES, Optima 8300, Perkin Elmer, Waltham, MA, USA).

The electrical conductivity (EC) was chosen as the soil salinity index. The leaching solution of 1:5 soil–water ratio was prepared according to the method described by the American Saline Soil Laboratory [49]. The soil pH was used to judge the acidity and alkalinity of the soil, and the ratio of soil to water was 1:5. The pH of the extract was determined using a PHS-3F pH meter (Shanghai Lei Magnetic Science Instrument Factory). Organic carbon concentrations in plants and soil were measured with the Walkley–Black method. Total nitrogen concentrations in plants and in soil were measured with the semi-micro-Kjeldahl method. Molybdenum antimony colorimetry was used to measure total phosphorus in plants and in soil.

### 4.3. Data Analysis

We calculated the descriptive statistics (mean, standard error and coefficient of variation) of the leaf, stem and root element concentrations in eight halophyte species of contrasting salt tolerance types (salt-dilution halophytes and recretohalophytes). A two-way ANOVA was performed to assess the effects of salt tolerance type, plant tissue parts and their connection to element concentrations. ANOVA was adopted to analyze the effect of different species on element concentrations. When species effects were significant (*p* < 0.05), we used Tukey’s HSD post hoc test to compare the means of the species. Pearson correlation coefficients were used to compare the correlations of elements among the leaf, stem, root and soil properties, as well as between the plant and soil. Differences in the elemental composition of the plant parts within soil salinity and soil mineral element were examined with redundancy analyses (RDA).

A general linear model (GLM) was employed to explore the variance in element concentrations in the leaves, stems and roots depending on salt tolerance type, soil salinity property and soil mineral elements. The total variance for each element was separated into salt tolerance type (species), soil salinity (pH and EC) and soil mineral element factors. All factors included in the linear model were assigned as independent factors. All data were log-transformed to normalize the distribution of element concentrations among leaves, stems and roots. All analyses were conducted using the statistical software SPSS 19.0, JMP (v.10.0.0; SAS Institute, Cary, NC, USA). Pearson correlation analysis was further completed by R-3.5.2 “corrplot” software package. Redundancy analysis uses Canoco5 for analysis.

## 5. Conclusions

The halophyte has higher element concentrations in the leaves than in the stems and roots. The higher concentrations of Na in salt-dilution halophytes than in recretohalophytes indicated that Na arguably plays an equal or more important role than K in osmoregulation. The variation of element concentrations within different organs was mainly determined by salt tolerance type and soil mineral elements. In summary, it can be concluded that element composition patterns in these studied Chenopodiaceae plants are the consequence of complex plant–soil interactions. These conclusions can provide an effective reference for evaluating plant breeding and its response to environmental change in saline–alkali arid areas in the Hulunbuir grassland, as well as other parts of the world.

## Figures and Tables

**Table 1:** Concentrations of elements in leaves, stems and roots of halophytes by salt tolerance type.

Elements (mg·g^−1^)	Salt-Dilution Halophyte			Recretohalophyte		
	Leaf	Stem	Root	Leaf	Stem	Root
P	1.07 ± 0.07 a	1.07 ± 0.08 a	0.89 ± 0.13 a	1.26 ± 0.14 a	1.19 ± 0.11 a	0.91 ± 0.1 a
N	18.87 ± 1.2 a	11.69 ± 0.65 b	7.99 ± 0.62 c	19.08 ± 1.04 a	13.63 ± 0.75 b	9.81 ± 0.69 c
C	344.01 ± 16.27 c	418.66 ± 15.49 b	485.57 ± 13.88 a	371.94 ± 14.92 b	436.67 ± 29.84 ab	501.75 ± 40.58 a
K	21.4 ± 3.65 a	18.19 ± 4.38 a	15.88 ± 3.22 a	29.12 ± 4.82 a	26.1 ± 4.83 a	23.62 ± 4.47 a
Ca	5.83 ± 1.25 a	4.59 ± 1.47 a	2.64 ± 0.17 a	9.35 ± 1.61 a	8.79 ± 2.58 a	5.73 ± 0.72 a
Mg	7.41 ± 0.5 a	4.55 ± 0.4 b	3.1 ± 0.36 c	9.75 ± 1.13 a	4.81 ± 0.55 b	5.95 ± 1.36 b
Na	72.26 ± 8.47 a	40.57 ± 5.94 b	24.08 ± 5.85 b	38.64 ± 10.23 a	18.27 ± 5.33 b	10.81 ± 2.96 b
Fe	1.15 ± 0.25 a	0.98 ± 0.18 a	1.33 ± 0.3 a	1.12 ± 0.32 a	0.73 ± 0.18 a	1.25 ± 0.24 a
Cu	0.01 ± 0 a	0.01 ± 0 a	0.07 ± 0.06 a	0.01 ± 0 a	0.01 ± 0 a	0.01 ± 0 a
Zn	0.02 ± 0 a	0.02 ± 0 a	0.01 ± 0 b	0.02 ± 0 a	0.02 ± 0 a	0.01 ± 0 a
Mn	0.09 ± 0.01 a	0.06 ± 0.01 b	0.05 ± 0.01 a	0.07 ± 0.01 a	0.03 ± 0 b	0.06 ± 0.01 a
Na/K	5.02 ± 0.8 a	4.41 ± 1.51 a	2.16 ± 0.49 a	2.29 ± 0.71 a	1.11 ± 0.36 ab	0.52 ± 0.13 b

Different letters represent the significant difference in the distribution of elements in a single salt tolerance type among different tissue parts according to one-way ANOVA with Turkey’s HSD test (*p* < 0.05).

**Table 2:** Two-way ANOVA was performed to assess the effects of salt tolerance type, plant tissue parts and their connection to element concentrations.

Elements (mg·g^−1^)	Salt Tolerance Type		Part		Salt Tolerance Type * Part	
	F	*p*	F	*p*	F	*p*
P	1.43	0.23	3.531	**0.033**	0.294	0.746
N	3.126	0.080	61.601	***0.000***	0.555	0.576
C	1.380	0.243	19.752	***0.000***	0.043	0.958
K	4.903	**0.029**	0.822	0.443	0.000	1.000
Ca	9.688	***0.002***	3.097	**0.050**	0.077	0.926
Mg	10.149	***0.002***	21.717	***0.000***	1.935	0.150
Na	14.881	***0.000***	14.079	***0.000***	0.970	0.383
Fe	0.287	0.593	1.332	0.269	0.093	0.911
Cu	0.751	0.389	0.607	0.547	0.599	0.552
Zn	0.468	0.496	2.890	0.061	0.181	0.835
Mn	5.200	**0.025**	12.252	***0.000***	2.747	0.070
Na/K	10.329	***0.002***	2.872	0.062	0.378	0.686

*p* values are in bold when *p* < 0.05 and in italic when *p* < 0.01. * means “×”, indicating the interaction of two factors.

**Table 3:** Summary of the general linear models for the effects of salt tolerance type, soil salinity property and soil mineral element factors on the concentrations of mineral elements in leaves.

Leaf Element	Total Effects (r^2^, %)			
	Full	Salt Tolerance Type	Soil Mineral Element	Soil Salinity
P	68.5	5.5	52.7	10.3
N	29.4	0.5	20.6	8.3
C	26.2	4.3	19.5	2.4
K	41.9	5.2	33.9	2.8
Ca	45.5	9.1	35.9	0.5
Mg	56.8	13.5	40.7	2.6
Na	54	17.1	32.9	4
Fe	53.3	0.01	52.2	1.1
Cu	50.7	12.5	30.9	7.3
Zn	58.9	1.4	55.1	2.4
Mn	47.9	9.7	32.4	5.8

Soil salinity variables: pH and electrical conductivity of soil saturated extract (EC); soil mineral elements: P, N, C, K, Ca, Mg, Na, Fe, Cu, Zn and Mn.

**Table 4:** Details of studied sites, their location, geodesic coordinates, and altitude (m).

Sites	Location	Geodesic Coordinates	Altitude (m)
1	Xin Barag Youqi	48°49′12.7″ N, 116°51′40.83″ E	547
2	Xin Barag Youqi	48°27′50.29″ N, 117°15′21.43″ E	593
3	Xin Barag Youqi	48°20′55.06″ N, 117°52′11.22″ E	574
4	Xin Barag Zuoqi	48°15′9.14″ N, 118°25′15.04″ E	689
5	Xin Barag Zuoqi	48°16′43.55″ N, 118°3′44.56″ E	585
6	Xin Barag Zuoqi	48°48′40.02″ N, 118°50′6.88″ E	685

**Table 5:** The physical, chemical and biological properties of plants belong to Chenopodiaceae family (i.e., soil texture, EC, pH, organic carbon, total nitrogen and total phosphorus).

Salt Tolerance Type	Species	Sites	Organ of Salt Concentration	Soil Texture	EC	pH	C	N	P
Salt-dilution halophyte	*Suaeda salsa* (L.) Pall.	1, 3, 4	Vacuole	Calcareous chernozem	0.104	7.71	11.60	0.29	0.65
	*Suaeda glauca* (Bunge) Bunge	1, 2, 3, 6			0.147	8.3	14.38	0.37	0.32
	*Kalidium foliatum* (Pall.) Moq.	1			0.167	7.84	12.91	0.43	0.41
	*Salsola collina* Pall.	1, 3			0.169	7.71	9.48	0.22	0.26
	*Kochia scoparia* (L.) Schrad. var. sieversiana (Pall.) Ulbr. ex Asch. & Graebn.	1, 3			0.142	7.84	15.52	0.54	0.40
Recretohalophyte	*Atriplex patens* (Litv.) Iljin	1, 2, 4, 5	Salt gland	Calcareous chernozem	0.130	7.73	18.62	0.48	0.25
	*Atriplex sibirica* L.	1, 2, 4, 5			0.140	8.01	11.44	0.40	0.41
	*Chenopodium glaucum* L.	1, 2, 5, 6			1.25	7.74	17.97	0.58	0.59

## Data Availability

Shown in the attached Table S1.

## Supplementary Materials

The following are available online at https://www.mdpi.com/article/10.3390/plants11101288/s1, Table S1. Summary of the general linear models for the effects of salt tolerance type, salt, and soil mineral element factors on concentrations of mineral elements in stems. Table S2. Summary of the general linear models for the effects of salt tolerance type, salt, and soil mineral element factors on concentrations of mineral elements in roots.
