# Supplementary material for: Study on the Effects of Salt Tolerance Type, Soil Salinity and Soil Characteristics on the Element Composition of Chenopodiaceae Halophytes
Source: Plants (Basel). 2022 May 11;11(10):1288. doi: 10.3390/plants11101288 (PMC9144953; doi:10.3390/plants11101288)
Supplement: Supplementary file 1 [file plants-11-01288-s001.zip › plants-1697392-supplementary.pdf]

Table S1. Summary of the general linear models for the effects of salt tolerance type, salt, and soil mineral element factors on concentrations of mineral elements in stems.

Stem element	Total Effects (r^2, %)			
	Full	Salt Tolerance Type	Soil Mineral Element	Soil Salinity
P	43.9	2.4	41.2	0.3
N	44.5	10.7	32.8	1
C	35.3	1.2	33.5	0.6
K	65.7	4.3	57.4	4
Ca	39.4	7.2	20.8	11.4
Mg	58.3	0.5	50.6	7.2
Na	41.9	17.8	22.4	1.7
Fe	54.9	2.7	35.8	16.4
Cu	47.1	6.6	34.1	6.4
Zn	56	0.01	52.4	3.6
Mn	67.9	25.8	29.6	12.5

Salt variables: pH and EC; soil mineral element: P, N, C, K, Ca, Mg, Na, Fe, Cu, Zn and Mn.

Table S2. Summary of the general linear models for the effects of salt tolerance type, salt, and soil mineral element factors on concentrations of mineral elements in roots.

Root Element	Total Effects (r^2, %)			
	Full	Salt Tolerance Type	Soil Mineral Element	Soil Salinity
P	41.3	0.03	40.5	0.8
N	35.7	10.7	20.6	4.4
C	47.3	0.7	42.1	4.5
K	61	6.4	49.4	5.2
Ca	69.3	47.6	21.4	0.3
Mg	59.9	17.1	34.6	8.2
Na	61.8	8.5	52.4	0.9
Fe	40	0.1	29.4	10.5
Cu	43.5	2.1	41.1	0.3
Zn	54.6	1.4	35.7	17.5
Mn	49.7	1.3	31.2	17.2

Salt variables: pH and EC; soil mineral element: P, N, C, K, Ca, Mg, Na, Fe, Cu, Zn and Mn.
